# Within-species variability of antibiotic interactions in Gram-negative bacteria

**DOI:** 10.1128/mbio.00196-24

**Published:** 2024-02-23

**Authors:** Po-Cheng Tang, Dione L. Sánchez-Hevia, Sanne Westhoff, Nikolaos Fatsis-Kavalopoulos, Dan I. Andersson

**Affiliations:** 1 Department of Medical Biochemistry and Microbiology, Uppsala University, Uppsala, Sweden; McMaster University Department of Biochemistry & Biomedical Sciences, Hamilton, Ontario, Canada

**Keywords:** antibiotic, drug interactions, synergy, antagonism, bacteria

## Abstract

Treatments with antibiotic combinations are becoming increasingly important even though the supposed clinical benefits of combinations are, in many cases, unclear. Here, we systematically examined how several clinically used antibiotics interact and affect the antimicrobial efficacy against five especially problematic Gram-negative pathogens. A total of 232 bacterial isolates were tested against different pairwise antibiotic combinations spanning five classes, and the ability of all combinations in inhibiting growth was quantified. Descriptive statistics, principal component analysis (PCA), and Spearman’s rank correlation matrix were used to determine the correlations between the different combinations on interaction outcome. Several important conclusions can be drawn from the 696 examined interactions. Firstly, within a species, the interactions are in general conserved but can be isolate-specific for a given antibiotic combination and can range from antagonistic to synergistic. Secondly, additive and antagonistic interactions are the most common observed across species and antibiotics, with 87.1% of isolate-antibiotic combinations being additive, 11.6% antagonistic, and only 0.3% showing synergy. These findings suggest that to achieve the highest precision and efficacy of combination therapy, not only isolate-specific interaction profiling ought to be routinely performed, in particular to avoid using drug combinations that show antagonistic interaction and an expected associated reduction in efficacy, but also discovering rare and potentially valuable synergistic interactions.

**IMPORTANCE:**

Antibiotic combinations are often used to treat bacterial infections, which aim to increase treatment efficacy and reduce resistance evolution. Typically, it is assumed that one specific antibiotic combination has the same effect on different isolates of the same species, i.e., the interaction is conserved. Here, we tested this idea by examining how several clinically used antibiotics interact and affect the antimicrobial efficacy against several bacterial pathogens. Our results show that, even though within a species the interactions are often conserved, there are also isolate-specific differences for a given antibiotic combination that can range from antagonistic to synergistic. These findings suggest that isolate-specific interaction profiling ought to be performed in clinical microbiology routine to avoid using antagonistic drug combinations that might reduce treatment efficacy.

## INTRODUCTION

Antibiotic resistance is a serious threat to modern medicine and global public health, and the slow rate of discovery of new antibiotics, together with the spread of resistance, threatens to undermine future options of antibiotic therapy (1). To safeguard the available antibiotics and ensure their effective and sustainable use in modern medicine, reducing inappropriate use of antibiotics and finding strategies to optimize the use and efficacy of our existing antibiotics are important public health priorities (2).

Antibiotic combination therapy is a common treatment strategy, for example, in hospital settings (3–6) and for tuberculosis treatment (7, 8). The reasons for using a combination can vary depending on the infection but at least five justifications can be identified: (i) to broaden the empirical coverage provided by several antibiotics with different spectra of activity in an effort to ensure the pathogen(s) is adequately covered by one of the drugs, (ii) to prevent/delay the emergence of resistance during treatment, (iii) to reduce the impact of existing resistance, (iv) to minimize drug toxicity in patients, and (v) to exploit antibiotic synergy to improve treatment outcome (6). With regard to the latter motivation, even though experimental studies have shown that certain antibiotic combinations may be synergistic (9–14), it still remains unclear if the synergies observed under these *in vitro* conditions are clinically exploitable and associated with an improved treatment outcome as compared to monotherapy (15). Conversely, it is also unclear as to what extent an antagonistic interaction observed in the laboratory translates into reduced efficacy during treatment. Thus, existing studies show diverse and conflicting results, often without any clear correlation between improved clinical outcome and use of antibiotic combinations (5, 6, 16–21). There are many potential explanations for this lack of correlation (9), but of relevance here is the occurrence of a general conservation of antibiotic interactions for a given species (11, 22) and any isolate-specific variation in the conserved antibiotic interaction patterns, which can obscure potential correlations to clinical outcome. A few previous studies have suggested that there indeed exists isolate variation (11, 22–24), but such studies are rare because existing testing technology, such as checkerboard and time-kill assays (25), is very resource demanding, effectively preventing the analysis and stratification of high numbers of clinical isolates.

Using a new method based on antibiotic diffusion in agar plates, CombiANT (23), we determined the occurrence of species and isolate-specific interaction patterns for clinical isolates across five Gram-negative pathogens, all of which belong to the problematic ESKAPE pathogens (26, 27). CombiANT makes use of inhibitory concentration of antibiotic-containing agar loaded into the CombiANT reservoirs. Applying an overlay of agar without antibiotics allows the antibiotic to diffuse into the surrounding overlaying agar, creating a linear concentration gradient landscape from inhibitory to no antibiotic, akin to the Kirby-Bauer disk diffusion susceptibility test. Given the specific geometries of CombiANT inserts, three reservoirs result in three overlapping concentration gradients, effectively creating three different pairwise concentration-combination landscapes. CombiANT has been validated against standard assays for assessing antibiotic interactions such as time-kill and checkerboard assays. It has been demonstrated to be easier to conduct compared to time-kill methods, and it avoids the intrinsic reproducibility errors of a microdilution-based checkerboard assay. To quantify the interactions, we used the fractional inhibitory concentration index (FICi), which applies the popular Loewe additivity model (9, 28, 29), as the common metric to allow comparisons within and between the different species. The model is based on the hypothesis that a drug cannot interact with itself and, therefore, the effect of a self-drug combination will always be additive, with an FICi = 1.

A panel of clinically relevant pairwise combinations spanning five different antibiotics classes (aminoglycosides, β-lactams, polymyxins, quinolones, and tetracyclines) was tested against 232 clinical isolates. We categorized the interaction profile for each antibiotic combination as additive, antagonistic, or synergistic for each of these isolates. Our results show that antibiotic interaction patterns are generally conserved within a species with important exceptions, and that the vast majority of the isolates from all five species exhibited either an additive or an antagonistic interaction. These findings demonstrate the need for isolate-specific testing of the efficacy of antibiotic combinations to achieve correct treatment of each isolate causing bacterial infection.

## MATERIALS AND METHODS

### Isolates, growth conditions, and culture media

All isolates were cultivated using either Muller-Hinton II (MH-II, BBL, Becton Dickinson) agar for solid cultures or cation-adjusted MH-II broth as liquid cultures. As liquid cultures, all incubations were performed with orbital shaking (198 rpm) at 37°C, unless otherwise specified. As cultures on agar, all incubations were performed overnight (18 ± 1 h) at 37°C in the dark. A total of 515 clinical isolates were purified from clinical samples and frozen at −80°C as 10% (vol/vol) Dimethyl sulfoxide (DMSO) stocks (cat. no. D8418, Merck). One hundred isolates were collected for each species (*Acinetobacter baumannii*, *Enterobacter cloacae*, *Escherichia coli*, *Pseudomonas aeruginosa*, *Klebsiella pneumoniae*) with the exception of *P. aeruginosa*, which had 115 isolates collected. The isolates were acquired from several sources: Uppsala University Hospital, Karolinska University Hospital and the Public Health Agency in Sweden, Technical University of Denmark in Denmark, and Emory University Hospital in the USA.

### Antibiotics and stocks

All antibiotics (ciprofloxacin, colistin, gentamicin, meropenem, piperacillin, and tobramycin) were purchased from Merck. Tigecycline was purchased through Apoteket (Swedish state-owned pharmaceuticals retailer) as Tygacil (Wyeth, Pfizer). All work and incubations with tigecycline were performed protected from light to prevent degradation of the antibiotic. All antibiotics were prepared as liquid stock solutions in their respective solvent (Table S1) and were stored at −20°C in polypropylene 1.5-mL reaction tubes (cat. no. 72.690.001, Sarstedt) as small aliquots for single-thaw use, except for meropenem and piperacillin, which were prepared fresh on the day of experiment. Tigecycline was frozen in 1.5-mL LightSafe micro centrifuge tubes (cat. no. Z688312, Merck), and colistin was frozen in 3.5-mL soda glass vials (cat. no. 005-1970-3,5-R, Bergman Laboratories).

### Interaction profile testing

FICi was used as a standardized measurement of the interaction between two antibiotics for the 232 susceptible isolates. FICi was determined using the CombiANT (Rx Dynamics AB, Uppsala, Sweden) method (23). Optimal concentrations of antibiotics were determined as previously described to create inhibition zones of approximately 5–10 mm in the reference isolates of every species. This was done to maximize the assays’ dynamic range. Isolates were screened against the pairwise combination for interactions with the following antibiotics and all possible pairwise combination for the antibiotics: *A. baumannii* included colistin, piperacillin, and tigecycline; *E. cloacae* included ciprofloxacin, gentamicin, and tigecycline; *E. coli* included ciprofloxacin, gentamicin, and piperacillin; *K. pneumoniae* included colistin, gentamicin, and tigecycline; and *P. aeruginosa* included meropenem, piperacillin, and tobramycin. This method has been validated against time-kill and checkerboard assays in the two previous studies (23, 24).

One-milliliter overnight liquid culture of all 515 isolates was initiated with a scrape of the isolate from pure-streak agar cultures (corresponding to proportions of four to five colonies) and was incubated. Upon overnight growth, the liquid cultures were diluted 20-fold in 0.9% (wt/vol) NaCl. One hundred microliters of the dilution was then spread across the surface of the agar using four to five sterile glass beads on activated CombiANT plates containing the antibiotic combinations of interest designated for the isolate. Upon spreading of the culture dilution across the surface of the activated agar plates, a single colistin impregnated filter paper was placed on the surface of the agar when required (further described below). If the isolate showed growth within the reservoir and no inhibition zone, it was designated resistant towards the antibiotic. If the isolate showed resistance towards any one of the three antibiotics tested, it was excluded in further FICi determinations. A minimum of three biological replicates was then repeated with different overnight cultures of the susceptible isolate, and the FICi was determined.

Due to thermal instability of colistin in agar at 55°C, we modified the method in order to examine the interactions with colistin. Whatman qualitative filter paper (grade 1, cat. no. WHA1001918, Merck) with the dimensions exactly to the size of the reservoir was impregnated with 30 µL of stock colistin solution followed by a 10-min air drying at 37°C and allowed to rest at room temperature until required. Upon spreading of the culture dilution across the surface of the activated agar plates, colistin-impregnated filter papers were placed on the surface of the agar either on reservoir A (single), on reservoirs A and B (double), or on all three reservoirs (A, B, and C). The placement of colistin filter papers on different reservoirs yielded acceptable FICi with a mean range of 0.88 to 0.93 between the different placement reservoirs and FICi (Fig. S1). When we combined colistin-impregnated filter papers with other antibiotics as described for the method (where agar and antibiotics are mixed together and placed in the reservoirs), the FICi was also determined to be acceptable for our purposes (Fig. S2).

Several measures were put in place to ensure technical assay reproducibility between experiments. The FICi from CombiANT compares the inhibitory concentration of a drug alone to its concentration in combination with another drug. The assay measures both effects simultaneously on the same replicate and agar plate, analyzing the inhibition zones formed outside and inside the reservoirs, respectively. This methodology ensures that combination effects are always evaluated relative to their individual effects, mitigating any diffusion inconsistencies.

Furthermore, each antibiotic is calibrated using ATCC reference strains for every species (Table S2) to account for the interplay between bacterial growth and antibiotic diffusion. In each experiment, reference strains serve as quality control, and the physics model is adjusted to match the reference strain’s minimum inhibitory concentration (MIC) based on the inhibition zone edge. This calibration ensures consistent calculations despite experimental variability and diffusion gradients, anchoring them to the known MIC of the reference strain. If the quality control strain did not perform with expected interactions, the experiment was considered invalid.

The FICi for each isolate was then defined as the average of all the replicates tested for each isolate and the value used to score the interaction. Scoring was carried out based on previously agreed classifications (28, 30, 31), which resulted in two types of categories: (i) according to accepted clinical thresholds for FICi values, where FICi values of ≤0.5 were considered synergistic, FICi values of >0.5 but ≤4 were considered additive, and FICi values of >4 were considered antagonistic, and (ii) according to deviations from theoretical additivity (32, 33), where FICi values >1 were considered theoretically antagonistic, FICi values <1 were considered theoretically synergistic, and FICi values = 1 were considered absolute theoretical additivity.

### Statistical and data analyses

Descriptive statistics for all data are available in Table S3 to S18. All statistical analyses were performed in GraphPad Prism v.9.5 for macOS. Default analysis function for descriptive statistics was used to determine the standard deviation of the mean FICi values for a given isolate-combination and the median of the mean FICi values for each isolate. A one-sample *t*-test was determined using the corresponding median of the mean FICi values as the hypothetical value, and the significance level (alpha) was set to 0.05. PCA was performed on a standardized scale. The input was the FICi based on the 232-isolate collection for all species in this study. Principal components (PCs) were picked based on two approaches: (i) by performing a parallel Monte Carlo analysis (34) on randomized data and when eigenvalues were greater than the randomized data set at the 95% level they were selected, and (ii) by only the eigenvalues following Kaiser criterion (35, 36) in which values greater than 1 were selected. Spearman’s rank correlation matrix was determined to calculate the correlation coefficient of the FICi of one antibiotic combination and the FICi of the 14 other combinations based on the 232-isolate collection for all species in this study.

## RESULTS

### A high-throughput method for determining isolate-specific interaction patterns

The study material consisted of a total of 515 clinical isolates catalogued as independent and represented distinct clinical cases from the clinical databases in Sweden, Denmark, and the USA. Clinical isolates spanned five different Gram-negative species, namely, *A. baumannii*, *E. cloacae*, *E. coli*, *K pneumoniae*, and *P. aeruginosa*. A total of seven antibiotics (ciprofloxacin, cip; colistin, col; gentamicin, gen; meropenem, mer; piperacillin, pip; tigecycline, tgc; and tobramycin, tob), spanning five antibiotic classes (aminoglycosides, β-lactams, polymyxins, quinolones, and tetracyclines), were used to generate 12 pairwise antibiotic combinations. The combinations tested using the high-throughput CombiANT method were unique for each species. They were primarily chosen to represent combinations that are clinically used, have been suggested for clinical use, or have been shown *in vitro* to be effective against the respective species for therapeutic treatment (Table S19). A second criterion for selecting a combination was to investigate how the interaction profile between similar drugs changes across different species: *A. baumannii*, tgc + pip, tgc + col, pip + col; *E. cloacae*, cip + tgc, cip + gen, tgc + gen; *E. coli*, cip + pip, cip + gen, pip + gen; *K. pneumoniae*, tgc + col, tgc + gen, gen + col; and *P. aeruginosa*, mer + pip, per + tob, pip + tob. In terms of combinations, an overlap combination between species was a by-product in combination choices. In our collection of 515 isolates, we identified and excluded the isolates that had a MIC above the clinical breakpoint for the respective antibiotic (based on European Committee on Antimicrobial Susceptibility Testing (EUCAST) guidelines). This gave 50 (50 %) *A. baumannii*, 85 (85 %) *E. cloacae*, 80 (80 %) *E. coli*, 81 (81 %) *K*. *pneumoniae*, and 53 (46 %) *P*. *aeruginosa* isolates that had MIC values below the clinical breakpoints. Finally, from a random subset of these susceptible isolates (49 *A*. *baumannii*, 50 *E. cloacae*, 52 *E. coli*, 53 *K*. *pneumoniae*, and 28 *P*. *aeruginosa*), the interaction profile was determined for a total of 232 isolates (Fig. S3). Variability in isolate-combination replicates was lower than the confidence margins of the checkerboard assay (25) (i.e., fourfold difference in FICi) with all strain/replicates having a 95% CI of the mean within a 2log_2_.

### Additive and antagonistic interaction patterns are highly prevalent in Gram-negatives with clinically relevant antibiotic combinations

**Fig 1:**
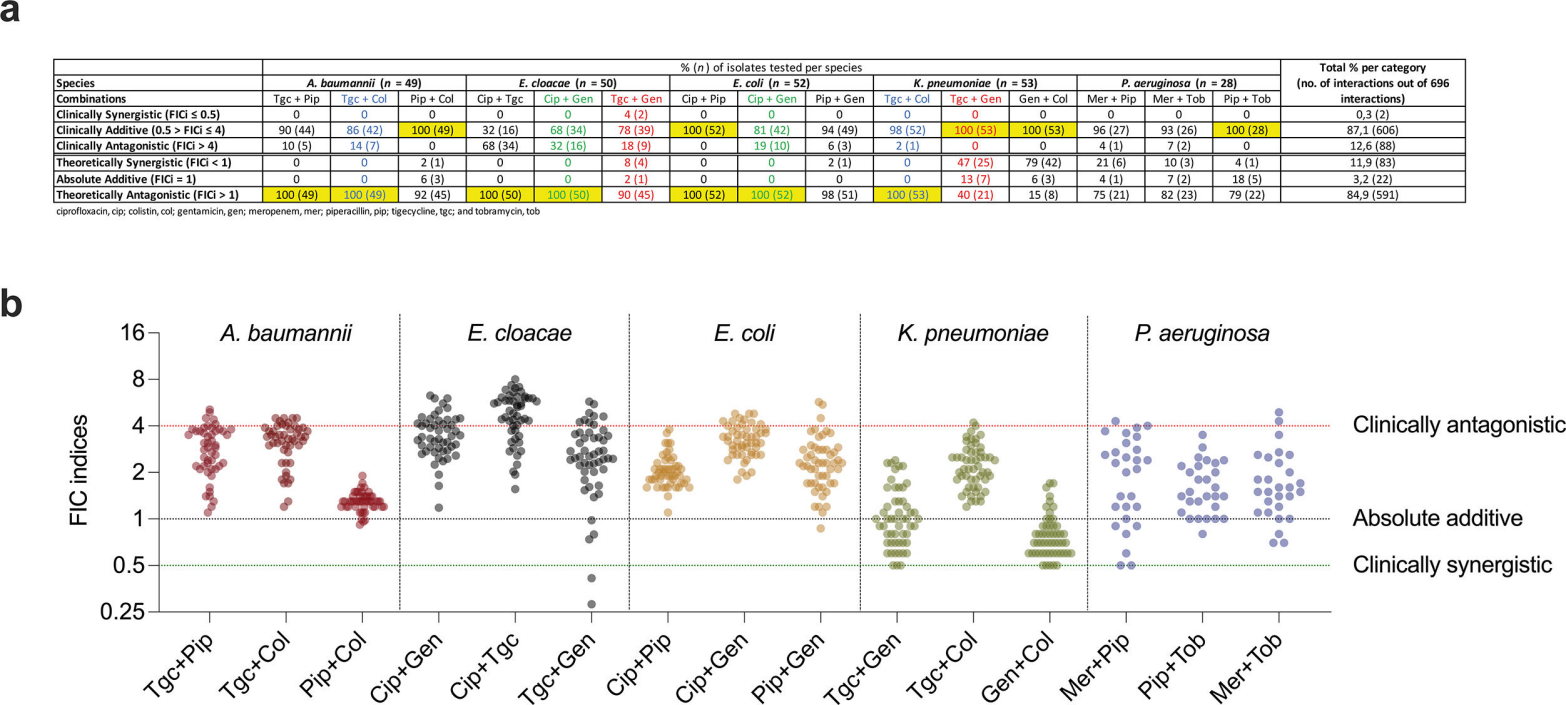
Antibiotic interactions of five Gram-negative pathogens against 12 clinically relevant combinations. (a) A table of antibiotic interactions for all combinations tested against the respective species. Combinations that were shared between different species are indicated in blue, green, and red, and combinations that resulted in 100% of the isolates for the category are indicated in yellow. Ciprofloxacin, cip; colistin, col; gentamicin, gen; meropenem, mer, piperacillin, pip; tigecycline, tgc; tobramycin, tob. (b) Fractional inhibitory concentration indices for all isolates across five species (*n* = 232) against 12 different pairwise antibiotic combinations in this study. Dotted lines at 0.5 (green), 1 (black), and 4 (red) indicate the clinical cutoffs for clinical synergy, absolute additivity, and clinical antagonism, respectively.

The interactions scored for 12 different antibiotic combinations against 232 Gram-negative isolates are summarized in Figure 1a. The average FICi values used to score all 12 different antibiotic combinations against 232 Gram-negative isolates are summarized as interaction pattern graphs in Figure 1b (described in detail as interaction profiles in Fig. S4 to S13; Table S20). Interaction can be categorized based on theoretical or clinical thresholds. In Fig. 1a, both definitions are included, and as can be seen depending on the cutoffs used, the fractions of the categories against a species change. For the rarely used theoretical categorization, synergy, additivity, and antagonism can simply be defined as FICi values <1, 1, or >1, respectively, where anything other than strict additivity is immediately classified as synergy or antagonism. Using the theoretical categorization, 84.9% of the isolates were antagonistic, and this was the most common pattern across all isolates, species, and antibiotic combinations (Fig. 1a). This was followed by 11.9% of the isolates being synergistic and only 3.2% being additive. Theoretical antagonism was observed in 100% of the isolates of *A. baumannii*, *E. cloacae*, *E. coli*, and *K. pneumoniae* against seven antibiotic combinations. Theoretical synergy was largely observed in *K. pneumoniae* and *P. aeruginosa* against five combinations, but also in small proportions in *A. baumannii*, *E. cloacae*, and *E. coli*. Theoretical additivity was observed for all species with the exception of *E. coli* and occurred in all the combinations tested for *P. aeruginosa*. It is important to note that there is no solid theoretical calculation of antibiotic interactions based on any model (Loewe additivity or Bliss independence) that does not take into account the noise of experimental data. Thus, FICi = 1 ± 2 standard deviations could be termed neutral interactions with 95% confidence on the condition of a normally distributed data set. Additionally, models (Loewe additivity or Bliss independence) have a null hypothesis. If most of the data do not satisfy the null hypothesis, either the data or the model is deemed wrong. It is important to consider the associated null hypothesis and the model used relating to the reported percentage of any antibiotic interactions.

The widely accepted clinical interaction categorization takes into account the impact of experimental errors and effect size. Synergy, additivity, and antagonism are defined as FICi values ≤0.5, >0.5 but ≤4, and >4, respectively, using the Loewe additivity model (25). For the clinical cutoffs, additive interactions account for 87.1% of the isolates and were the most common outcome across all isolates, species, and antibiotic combinations (Fig. 1). This was followed by clinical antagonism for 12.6% of the isolates, and only 0.3% of the isolates were categorized as clinically synergistic.

When examining the interaction patterns separately for each species, it is clear that even though there are species differences, additivity and antagonism are by far the most prevalent categories (Fig. 1, 2a and b) with a few exceptions (e.g., GEN + COL and TGC + GEN combinations against *K. pneumoniae*). For *A. baumannii*, *E. coli*, *K. pneumoniae*, and *P. aeruginosa*, all isolates showed either additivity or antagonism with no cases of synergy for the three antibiotic combinations tested. For *E. cloacae*, the pattern was slightly different with a higher fraction of clinical antagonism (observed for all three antibiotic combinations) but also synergy. However, the clinical synergy was observed in only two *E. cloacae* isolates (out of 50 isolates) and only against the tgc + gen combination. Interestingly, for this particular antibiotic combination, all types of interactions were observed among the 50 isolates (Fig. 1a).

**Fig 2:**
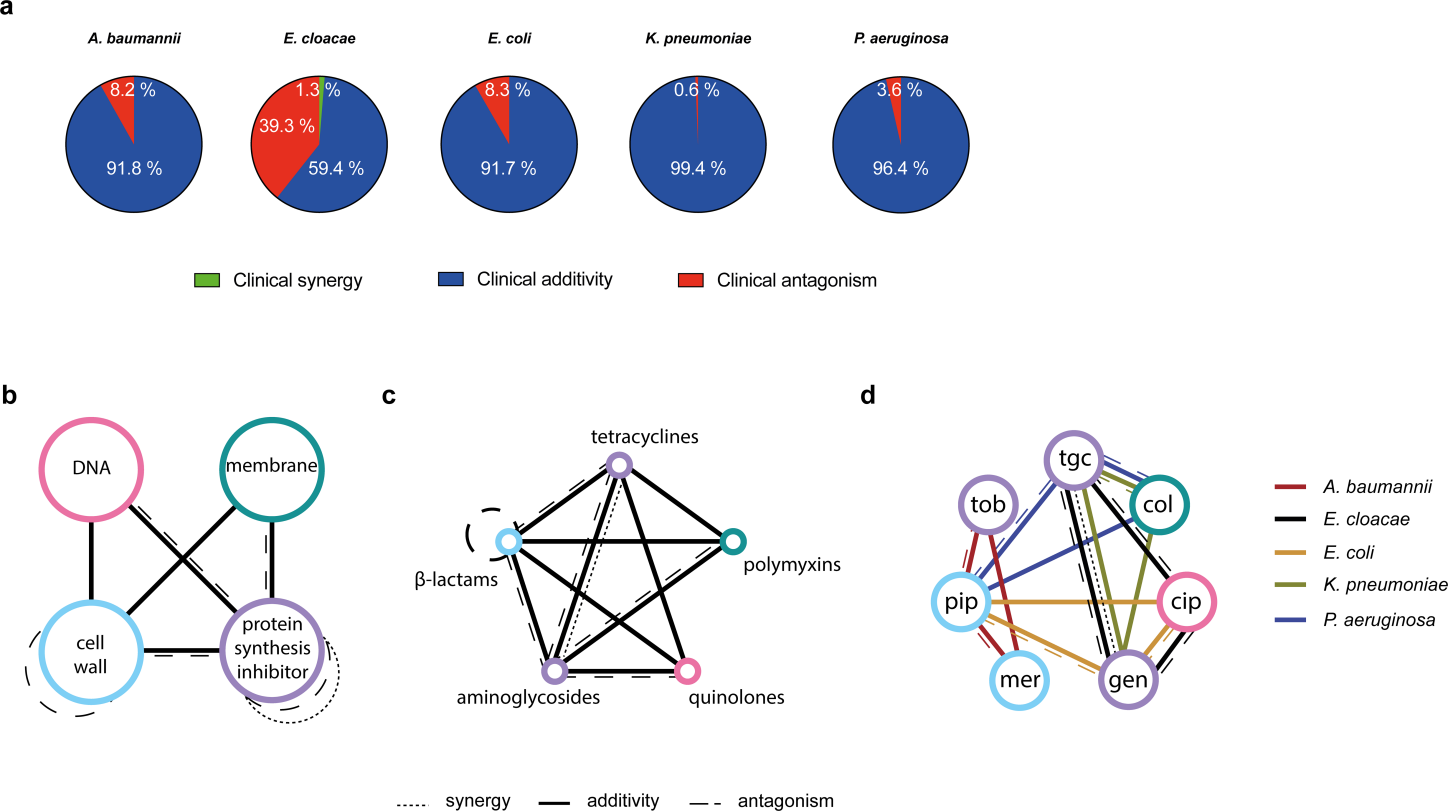
Interaction patterns and drug-drug networks in five Gram-negative bacterial species. (a)Each fraction of a pie represents the total percentage of the clinical categories (synergy, additivity, or antagonism) determined for the three combinations tested against each species. (b)Network on the basis of the drug’s cellular process target, (c)network on the basis of the drug’s class, and (d)network on the basis of the drugs. Nodes with different colors represent grouping based on the general cellular process the drug targets. Different line styles (solid, dashed, and dotted) denote the clinical interaction categories observed based on Fig. 1a (additivity, antagonism, and synergy, respectively). Different line colors denote the different species. Different colored circles represent the antibiotics and their corresponding drug target and drug class.

In summary and most importantly, based on FICi thresholds, additive and antagonistic interaction patterns were the most prevalent, and synergy was rare, with this response only found in 0.3% of the isolates using the more conservative clinical definition.

### Intra- and inter-species correlations between antibiotic combinations and their interaction patterns

**Fig 3:**
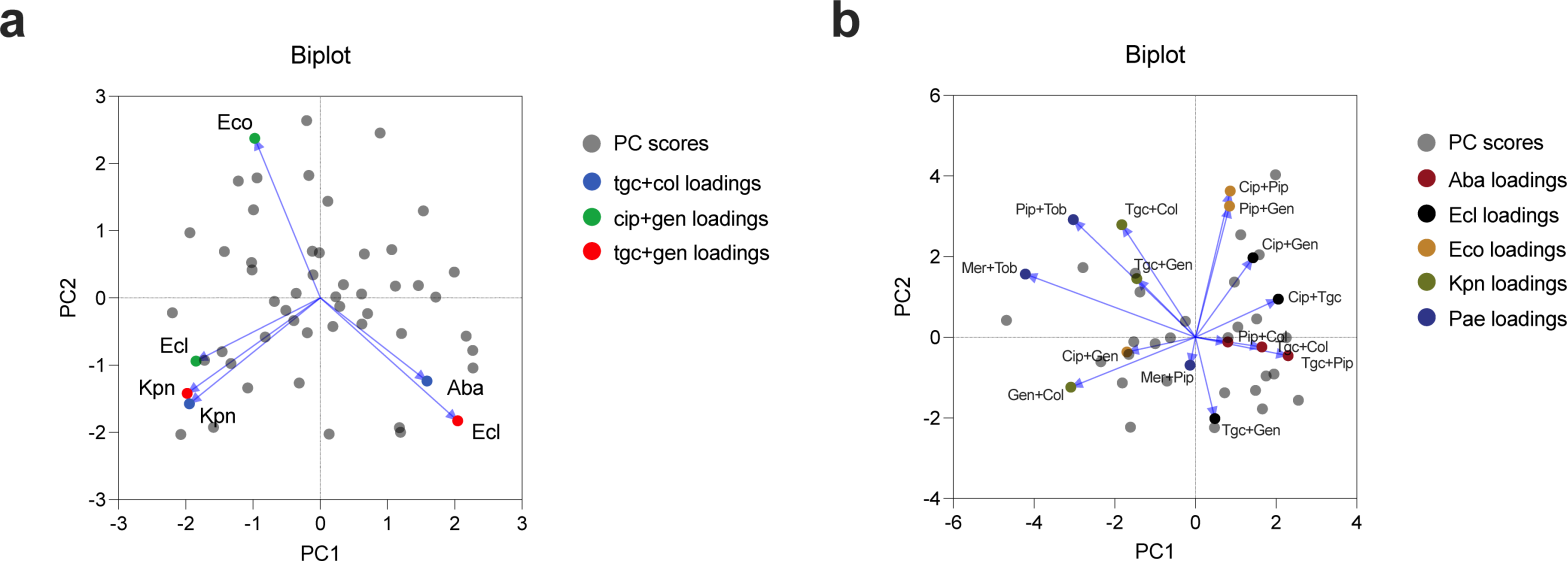
Growth inhibitory effects of antibiotic combinations. (a) PCA biplot of multiple combinations tested in more than one species; gray dots denote individual PC scores; different colored loadings with blue arrows denote the shared combinations tested in more than one species. (b) PCA biplot of the interaction patterns tested for each species; gray dots denote individual PC scores; different colored loadings with blue arrows denote the species in which the antibiotic combination was tested for comparison. *A. baumannii*, Aba; *E. cloacae*, Ecl; *E. coli*, Eco; *P. aeruginosa*, Pae; *K. pneumoniae*, Kpn.

Next, we set out to investigate if every antibiotic combination results in a distinct interaction pattern or whether there are combinations that are similar. This was carried out in two ways: first, between the same combinations used on different species to investigate possible species-crossing effects in multiple Gram-negative organisms, and second, between different combinations used on the same species to investigate if there are similar responses to different combinations within one organism. There were multiple combinations that were tested in more than one species (Fig. 1). We performed PCA on the interaction pattern using the average FICi values for all isolates in every combination that was tested across multiple species. For none of the species did the coefficients of the linear combination of the original variables from which the PCs are constructed (loadings) cluster in any combination, indicating that different species do not exhibit a similar response in interaction pattern to treatment with the same antibiotic combination (Fig. 3a; Fig. S14A and B). To investigate if the same species displays similar interaction patterns to different antibiotic combinations, we performed PCA on the average FICi values for all isolates in all combinations tested (Fig. 3b; Fig. S14C and D). Generally, the loadings of the different combinations did not seem to cluster, suggesting independent interaction pattern outcomes of every combination. There were, however, a few notable exceptions. All three combinations tested on *A. baumannii* clustered together as well as the combinations of cip + pip and pip + gen tested against *E. coli*. In the case of *A. baumannii,* tgc and col are present in both clustering combinations, and in the case of *E. coli*, the common drug is pip. The observations of the PCA were verified by a post-hoc Spearman’s rank correlation coefficient test. Only the correlation between the tgc + col and tgc + pip combinations in *A. baumannii* was verified to be extensive and significant (69% correlation, *P* < 0.001).

## DISCUSSION

The primary strength of our study is the extensive data set involving various isolates and antibiotic combinations. This broad scope allowed us to observe and report on a wide range of interaction patterns, offering valuable insights of combination therapy and potentially contribute to a more informed selection of antibiotic combinations in clinical settings. Despite these strengths, our study also has limitations that warrant consideration. Firstly, our analysis was confined to *in vitro* conditions, which, although insightful, might not fully replicate the intricacies of *in vivo* environments where patient-specific factors and other variables could influence antibiotic efficacy and interactions. Therefore, the direct translation of our findings to clinical practice should be approached with caution. Secondly, our focus was limited to five Gram-negative pathogens. Although these pathogens are clinically significant, our findings might not be generalizable to other bacterial species, limiting the broader applicability of our results.

In spite of these limitations, several important conclusions can be drawn from the presented results. First, and most important, within a species and for a given antibiotic combination, there can be extensive variation between isolates ranging from antagonistic to additive to synergistic (e.g., for *E. cloacae* with the tgc + gen combination). Second, although not exhaustive by any means, the interaction pattern for a given antibiotic combination can vary between different Gram-negative species (see for example, tgc + gen in *E. cloacae* and *K. pneumoniae*), precluding any general extrapolations between species. However, such conservation studies have been reported previously (11, 22, 37). Third, additive and antagonistic interactions are by far the most common patterns observed across species and antibiotics in which 99.7% (694/696) of all tested isolates and antibiotic combinations belonged to either of these two types, with as many as 12.6% of the isolates showing antagonistic interactions. Fourth, when synergy was observed, it was always restricted to a few isolates (2/696, 0.3% of all isolates), and we did not find any species and antibiotic combination in which all the isolates consistently showed synergy. These percentages reflect the clinical therapeutic scenarios with regard to these antibiotic combinations.

Additivity or antagonism is generally observed together for all drug classes and antibiotic targets, according to the drug-drug interaction networks we generated (Fig. 2b through d), and the fraction of additivity and antagonism is much higher than those reported in other studies (11, 22, 38). There are several potential explanations for these differences associated with experimental setup and how the interactions are quantified. One main difference is that in previous works, the number of isolates from a given species is often small (often only one or a few isolates are tested), suggesting that stochastic effects based on small strain selections can bias the overall picture of an interaction. Based on our findings, we suggest that testing of a large number of isolates of one species is essential to obtain a comprehensive picture and that generalizing to an entire species based on interactions from one or a few isolates (often lab-adapted strains) might be problematic from a clinical standpoint because isolate-specific antagonistic interactions might go undetected (38–41). A second important difference is the growth reduction quantification used in other studies to assess the combination effects, which is counter to the methodology used worldwide for single antibiotic testing, as the output measured is always the inhibitory capacity of an antibiotic and not the partial reduction in growth. Finally, the model applied to score interactions in several cases is the Bliss independence model, which is fundamentally different from the Loewe model used in this and other studies (42).

It is also of note that our study uses the FICi thresholds of 4 and 0.5 for antagonism and synergy, which were previously established. These thresholds, although well used, do not represent a clear link between FICi limits and clinical outcome. Therefore, one cannot truly classify a drug interaction as clinically antagonistic or synergistic, especially because intra-strain variability might still land a strain on either side of the imposed thresholds. Species-specific patterns, however, can still be observed like the higher prevalence of antagonistic interactions in *E. cloacae* compared to the other species.

We attempted to compare the combinations tested here with those in previous studies, but the majority of clinical and *in vitro* literature examines single- or multi-antibiotic-resistant bacteria, making direct intra-species variation in antibiotic-susceptible bacteria comparisons difficult. Furthermore, a majority of the clinical studies, where outcome is compared for monotherapy and combination therapy, lack a corresponding stratification of the isolates with regard to their *in vitro* interaction profiles. The latter is a major concern because in clinical studies, positive effects due to synergy for some isolates might be masked by antagonism in other isolates (and vice versa), making interpretation of outcomes problematic. Thus, future studies of treatment outcomes need to be directly associated with the *in vitro* interaction profile of the isolate derived from the patient (43).

This study focused on isolates that were susceptible towards all the antibiotics that were tested for that particular species, i.e., any isolate with a MIC above the clinical breakpoint at the time of testing was excluded from the analysis. The rationale for only examining susceptible isolates was based on three considerations. Firstly, antibiotic treatment generally excludes antibiotics to which the bacteria are resistant, once diagnostic information becomes available. Secondly, different resistance mechanisms confer resistance towards different antibiotics. Consequently, the interaction profile for a combination of antibiotics could vary due to the differences between the resistance mechanisms (24). Thirdly, not all molecular mechanisms responsible for antibiotic resistance towards a single antibiotic are known, and, if they were, it is not clear that all mutations would be relevant in the observed interaction profiles for the species. Thus, antibiotic-resistant isolates of a species with several resistance mechanisms, without the initial examination of susceptible bacteria of the same species, would not represent a homogenous population in which species-specific trends can be determined.

The most important implication from our results regards the common use of empirical antibiotic combination therapy in clinical settings. To prevent a potential reduction in efficacy, drug combinations and isolates that show antagonistic interactions (12.6% in this study) would seem important to avoid, and conversely, identification of the isolates where synergy is observed could potentially increase treatment efficacy in those rare cases. However, from a clinical treatment standpoint, it is probably more important to avoid antagonism than to identify synergy. This can only be achieved by isolate-specific interaction testing as part of the clinical microbiology routine (23). Furthermore, because many of the antibiotics included in this study are used for treatment of patients in hospital settings, who are more likely to be infected with resistant bacteria, an important next step would be to systematically investigate the interaction profiles of isolates that are partly or fully resistant to the individual antibiotics. This could result in the identification of the rare cases where combinations might overcome the resistance (44).

One central question (which was not examined in this study) is which genetic differences underlie the isolate-specific interaction patterns. At present, this is not known at all but it could potentially be addressed, for example, by association studies where genetic differences (DNA sequence [45] or gene expression [46]) between the isolates are associated to the interaction categories or by isolation of mutants with an altered interaction profile (14, 47, 48).

In addition to these genetic considerations, understanding the mechanistic basis for observed antagonism in antibiotic combinations is crucial. For instance, with tigecycline, a bacteriostatic agent, its growth inhibition property may interfere with the efficacy of other bactericidal antibiotics that rely on active growth. This interplay between the modes of action of different antibiotics is a crucial vaulting point for further mechanistic research and a critical aspect of antibiotic interactions that warrants further exploration (49).

## Data Availability

All data used to draw the conclusions in this paper are provided in the paper and/or in the supplementary materials.

## SUPPLEMENTAL MATERIAL

Supplemental figures mbio.00196-24-s0001.pdf Figures S1–S14.

Supplemental tables mbio.00196-24-s0002.xlsx Table S1–S20.

ASM does not own the copyrights to Supplemental Material that may be linked to, or accessed through, an article. The authors have granted ASM a non-exclusive, world-wide license to publish the Supplemental Material files. Please contact the corresponding author directly for reuse.
