# Supplementary material for: Within-species variability of antibiotic interactions in Gram-negative bacteria
Source: mBio. 2024 Feb 23;15(3):e00196-24. doi: 10.1128/mbio.00196-24 (PMC10936430; doi:10.1128/mbio.00196-24)
Supplement: Supplemental figures — Figures S1–S14. [file mbio.00196-24-s0001.pdf]

Within-species variability of antibiotic interactions in Gram-negative bacteria

Po-Cheng Tang, Dione L. Sánchez-Hevia†, Sanne Westhoff†, Nikos Fatsis-Kavalopoulos†, Dan I. Andersson*

†These authors contributed equally to this manuscript.

*Corresponding author. E-mail: dan.andersson@imbim.uu.se

Supplementary Figures 1-14 and legends

Legends Supporting Figures

Fig S1. Evaluation of modified CombiANT® method for use with colistin. a) colistin impregnated filter paper placed in 3 different reservoirs on the CombiANT® plates. b) mean and standard error of the mean of the obtained FIC indices in a.

Fig S2. Evaluation of modified CombiANT® method for use with colistin together with other antibiotics for all Gram-negative species in this study. The quality control strains for each species were tested. Antibiotics tested against the species are indicated followed by the reservoir they occupied on the CombiANT® plate. Top row of graphs (a, b, c, d, e) indicates the FICi obtained for three replicates against the pairwise combination of antibiotics. Bottom row of graphs (f, g, h, i, j) indicates the mean and standard error of the mean of the FICi obtained for the top row of graphs.

Fig S3. Schematic overview of this study. 232 Gram-negative clinical isolates were tested against seven different antibiotics resulting in 12 unique pair-wise combinations and 696 interaction profiles quantified.

Fig S4. Distribution of *A. baumannii* antibiotic interaction FIC indices (FICi) (*n*=48). The x axis denotes the FIC indices (FICi), the y axis denotes the percentage of isolates observed. The red dotted lines represent the clinical level for antagonism (FICi=4), the green dotted lines represent the clinical levels for synergy (FICi=0.5), the black dotted lines represent the absolute additivity (FICi=1).

Fig S5. *A. baumannii* antibiotic interaction FIC indices (FICi) (*n*=48). The x axis for graphs in both columns denotes the isolate number. The y axis for the graphs on the left column denotes FICi values for each replicate. The y axis for the graphs on the right column denotes the mean FICi and standard error of the mean calculated based on the FICi on the left column. All mean FICi values calculated can be found in supplementary table 1. The red dotted lines represent the clinical level for antagonism (FICi=4), the green dotted lines represent the clinical levels for synergy (FICi=0.5), the black solid line represent the absolute additivity (FICi=1).

Fig S6. Distribution of *E. cloacae* antibiotic interaction FIC indices (FICi) (*n*=50). The x axis denotes the FIC indices (FICi), the y axis denotes the percentage of isolates observed. The red dotted lines represent the clinical level for antagonism (FICi=4), the green dotted lines represent the clinical levels for synergy (FICi=0.5), the black dotted lines represent the absolute additivity (FICi=1).

Fig S7. *E. cloacae* antibiotic interaction FIC indices (FICi) (*n*=50). The x axis for graphs in both columns denotes the isolate number. The y axis for the graphs on the left column denotes FICi values for each replicate. The y axis for the graphs on the right column denotes the mean FICi and standard error of the mean calculated based on the FICi on the left column. All mean FICi values calculated can be found in supplementary table 1. The red dotted lines represent the clinical level for antagonism (FICi=4), the green dotted lines represent the clinical levels for synergy (FICi=0.5), the black solid line represent the absolute additivity (FICi=1).

Fig S8. Distribution of *E. coli* antibiotic interaction FIC indices (FICi) (*n*=52). The x axis denotes the FIC indices (FICi), the y axis denotes the percentage of isolates observed. The red dotted lines represent the clinical level for antagonism (FICi=4), the green dotted lines represent the clinical levels for synergy (FICi=0.5), the black dotted lines represent the absolute additivity (FICi=1).

Fig S9. *E. coli* antibiotic interaction FIC indices (FICi) (*n*=52). The x axis for graphs in both columns denotes the isolate number. The y axis for the graphs on the left column denotes FICi values for each replicate. The y axis for the graphs on the right column denotes the mean FICi and standard error of the mean calculated based on the FICi on the left column. All mean FICi values calculated can be found in supplementary table 1. The red dotted lines represent the clinical level for antagonism (FICi=4), the green dotted lines represent the clinical levels for synergy (FICi=0.5), the black solid line represent the absolute additivity (FICi=1).

Fig S10. Distribution of *K. pneumoniae* antibiotic interaction FIC indices (FICi) (*n*=53). The x axis denotes the FIC indices (FICi), the y axis denotes the percentage of isolates observed. The red dotted lines represent the clinical level for antagonism (FICi=4), the green dotted lines represent the clinical levels for synergy (FICi=0.5), the black dotted lines represent the absolute additivity (FICi=1).

Fig S11. *K. pneumoniae* antibiotic interaction FIC indices (FICi) (*n*=53). The x axis for graphs in both columns denotes the isolate number. The y axis for the graphs on the left column denotes FICi values for each replicate. The y axis for the graphs on the right column denotes the mean FICi and standard error of the mean calculated based on the FICi on the left column. The red dotted lines represent the clinical level for antagonism (FICi=4), the green dotted lines represent the clinical levels for synergy (FICi=0.5), the black solid line represent the absolute additivity (FICi=1).

Fig S12. Distribution of *P. aeruginosa* antibiotic interaction FIC indices (FICi) (*n*=28). The x axis denotes the FIC indices (FICi), the y axis denotes the percentage of isolates observed. The red dotted lines represent the clinical level for antagonism (FICi=4), the green dotted lines represent the clinical levels for synergy (FICi=0.5), the black dotted lines represent the absolute additivity (FICi=1).

Fig S13. *P. aeruginosa* antibiotic interaction FIC indices (FICi) (*n*=53). The x axis for graphs in both columns denotes the isolate no. The y axis for the graphs on the left column denotes FICi values for each replicate. The y axis for the graphs on the right column denotes the mean FICi and standard error of the mean calculated based on the FICi on the left column. All mean FICi values calculated can be found in supplementary table 3. The red dotted lines represent the clinical level for antagonism (FICi=4), the green dotted lines represent the clinical levels for synergy (FIC=0.5), the black solid line represent the absolute additivity (FICi=1).

Fig S14. Principal component analysis of all species interaction profile against each other following the parallel Monte Carlo analysis. a) Eigen values calculated for each of the principal component (PC). b). The percentage proportion of variance calculated for each of the PC for the

individual and cumulative PC. PCA of the interaction profile from all combinations tested for the different species. c). Eigen values calculated for each of the PC. d). The percentage proportion of variance calculated for each of the PC for the individual and cumulative PC.

Supp Fig 1.

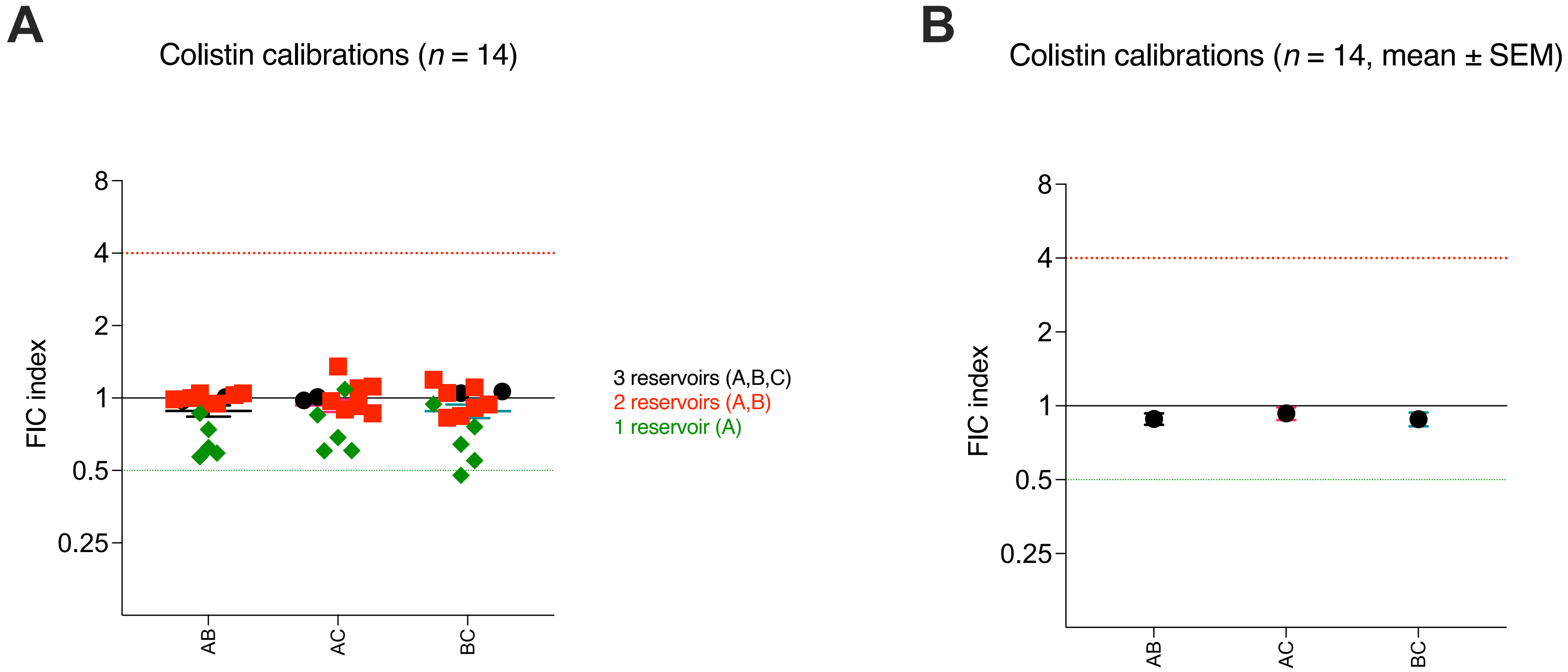

Supp Fig 2.

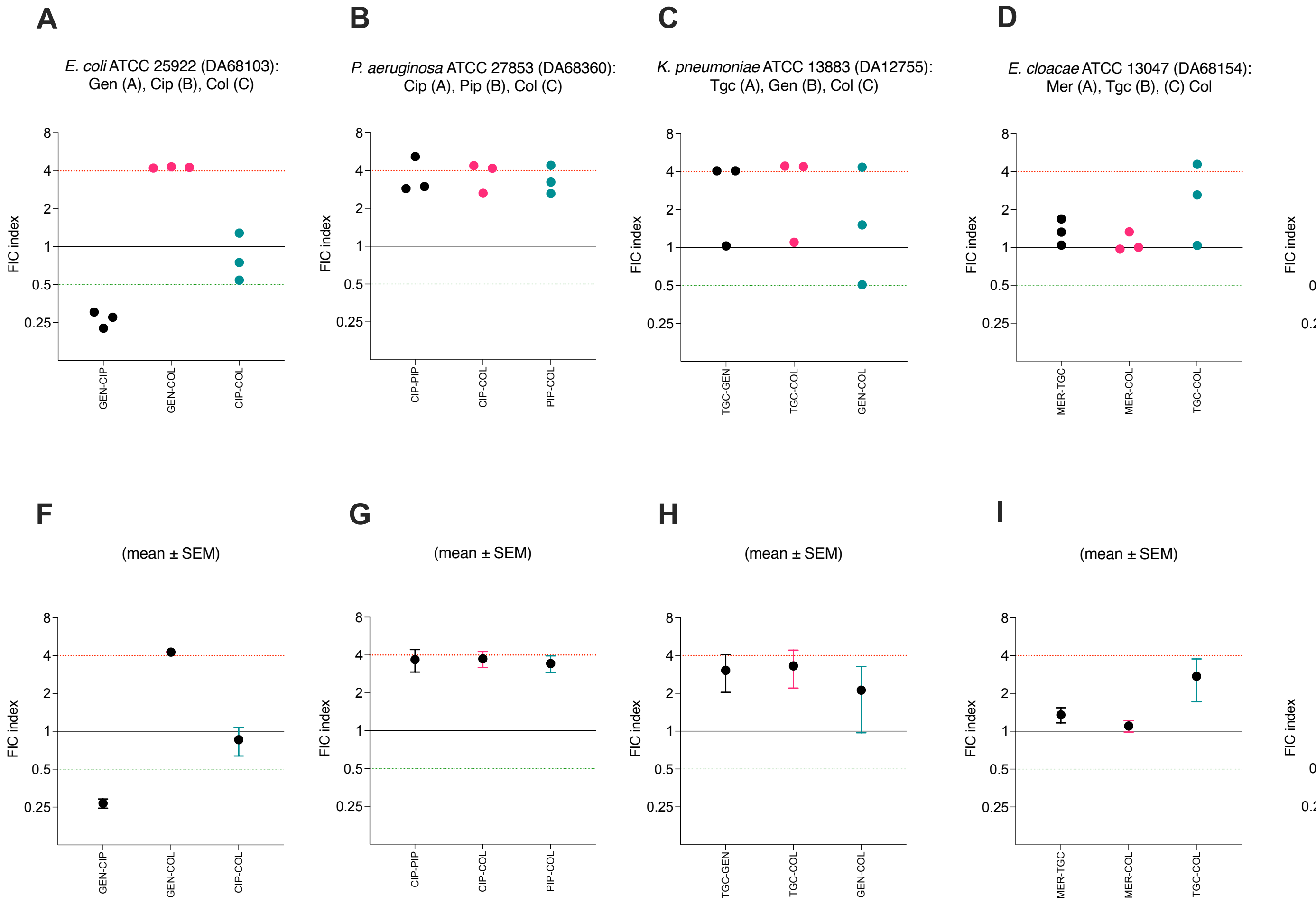
A
E. coli ATCC 25922 (DA68103):
Gen (A), Cip (B), Col (C)
FIC index
8
4
2
1
0.5
0.25
GEN-CIP
GEN-COL
CIP-COL
B
P. aeruginosa ATCC 27853 (DA68360):
Cip (A), Pip (B), Col (C)
CIP-PIP
CIP-COL
PIP-COL
C
K. pneumoniae ATCC 13883 (DA12755):
Tgc (A), Gen (B), Col (C)
TGC-GEN
TGC-COL
GEN-COL
D
E. cloacae ATCC 13047 (DA68154):
Mer (A), Tgc (B), (C) Col
MER-TGC
MER-COL
TGC-COL
F
(mean ± SEM)
G
H
I

Supp Fig 3.

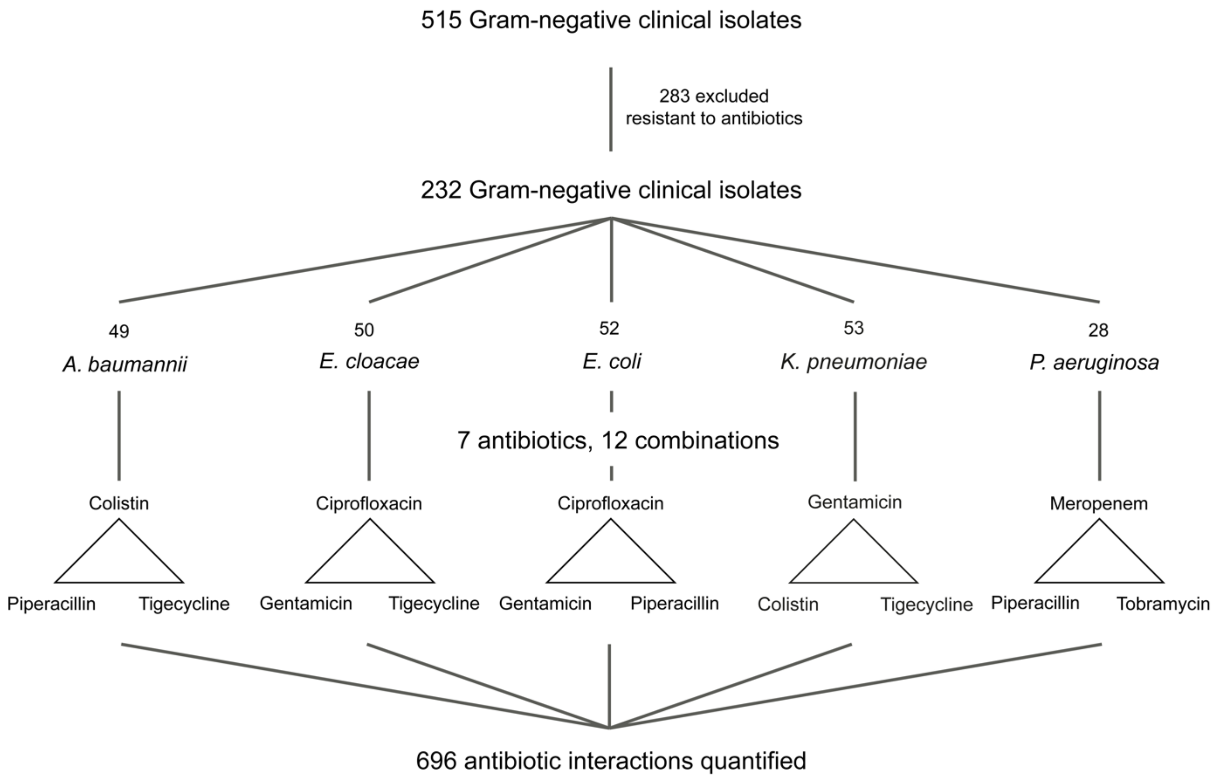
515 Gram-negative clinical isolates
283 excluded
resistant to antibiotics
232 Gram-negative clinical isolates
49
A. baumannii
50
E. cloacae
52
E. coli
53
K. pneumoniae
28
P. aeruginosa
7 antibiotics, 12 combinations
Colistin
Piperacillin
Tigecycline
Ciprofloxacin
Gentamicin
Tigecycline
Ciprofloxacin
Gentamicin
Piperacillin
Gentamicin
Colistin
Tigecycline
Meropenem
Piperacillin
Tobramycin
696 antibiotic interactions quantified

Supp Fig 4.

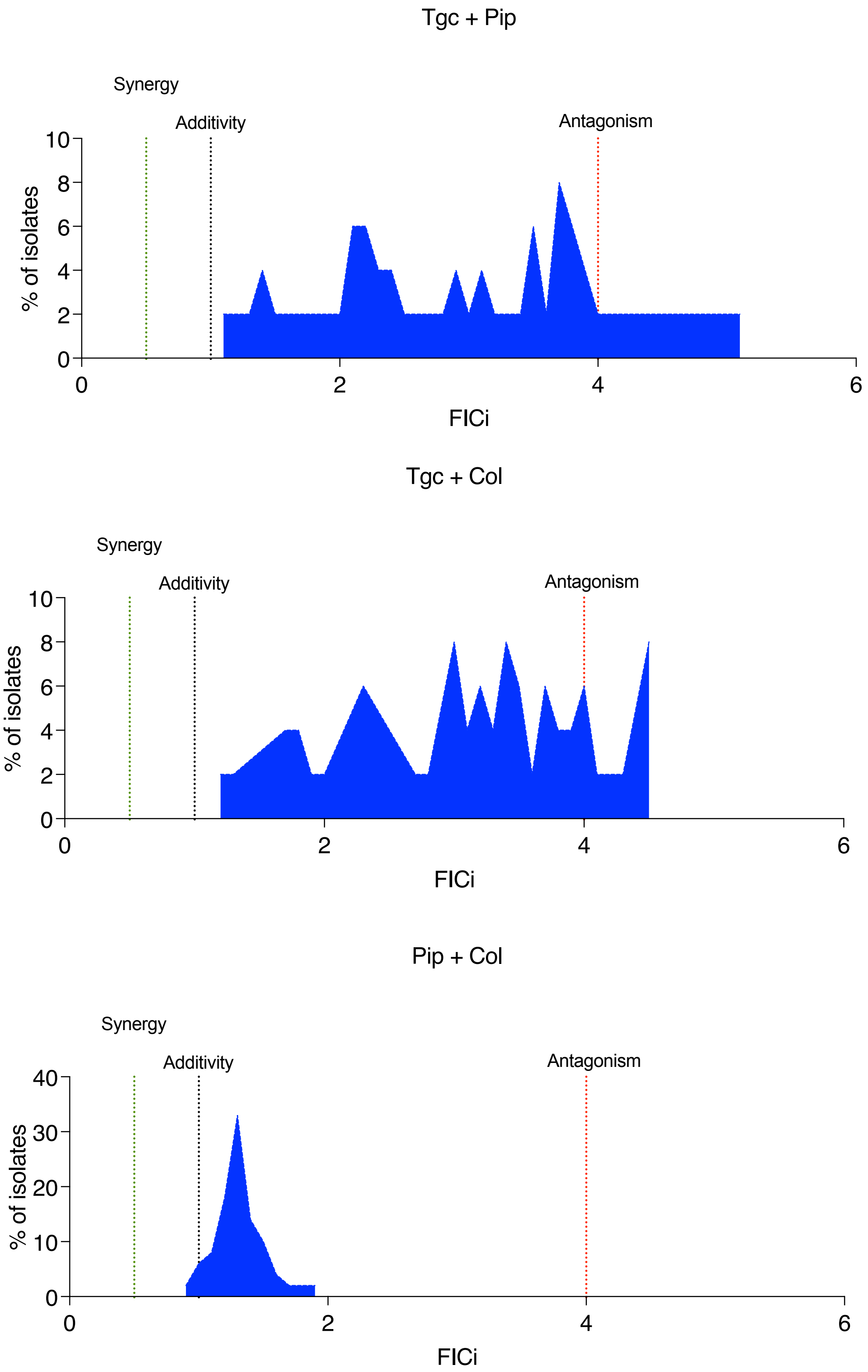
Tgc + Pip
Synergy
Additivity
Antagonism
% of isolates
0
2
4
6
8
10
0
2
4
6
FICi
Tgc + Col
Synergy
Additivity
Antagonism
% of isolates
0
2
4
6
8
10
0
2
4
6
FICi
Pip + Col
Synergy
Additivity
Antagonism
% of isolates
0
10
20
30
40
0
2
4
6
FICi

Supp Fig 5.

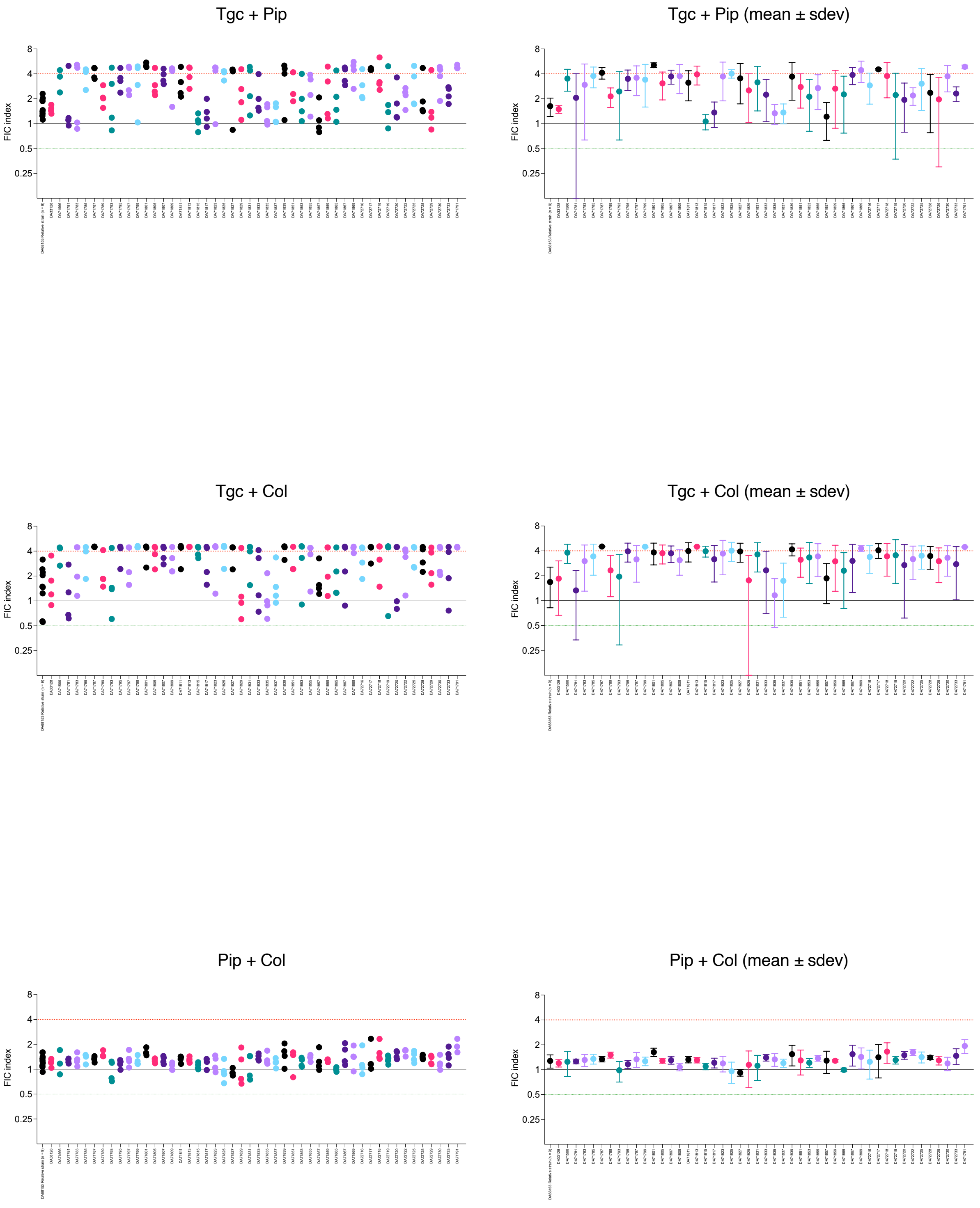
Tgc + Pip
Tgc + Pip (mean ± sdev)
Tgc + Col
Tgc + Col (mean ± sdev)
Pip + Col
Pip + Col (mean ± sdev)
FIC index
8
4
2
1
0.5
0.25

Supp Fig 6.

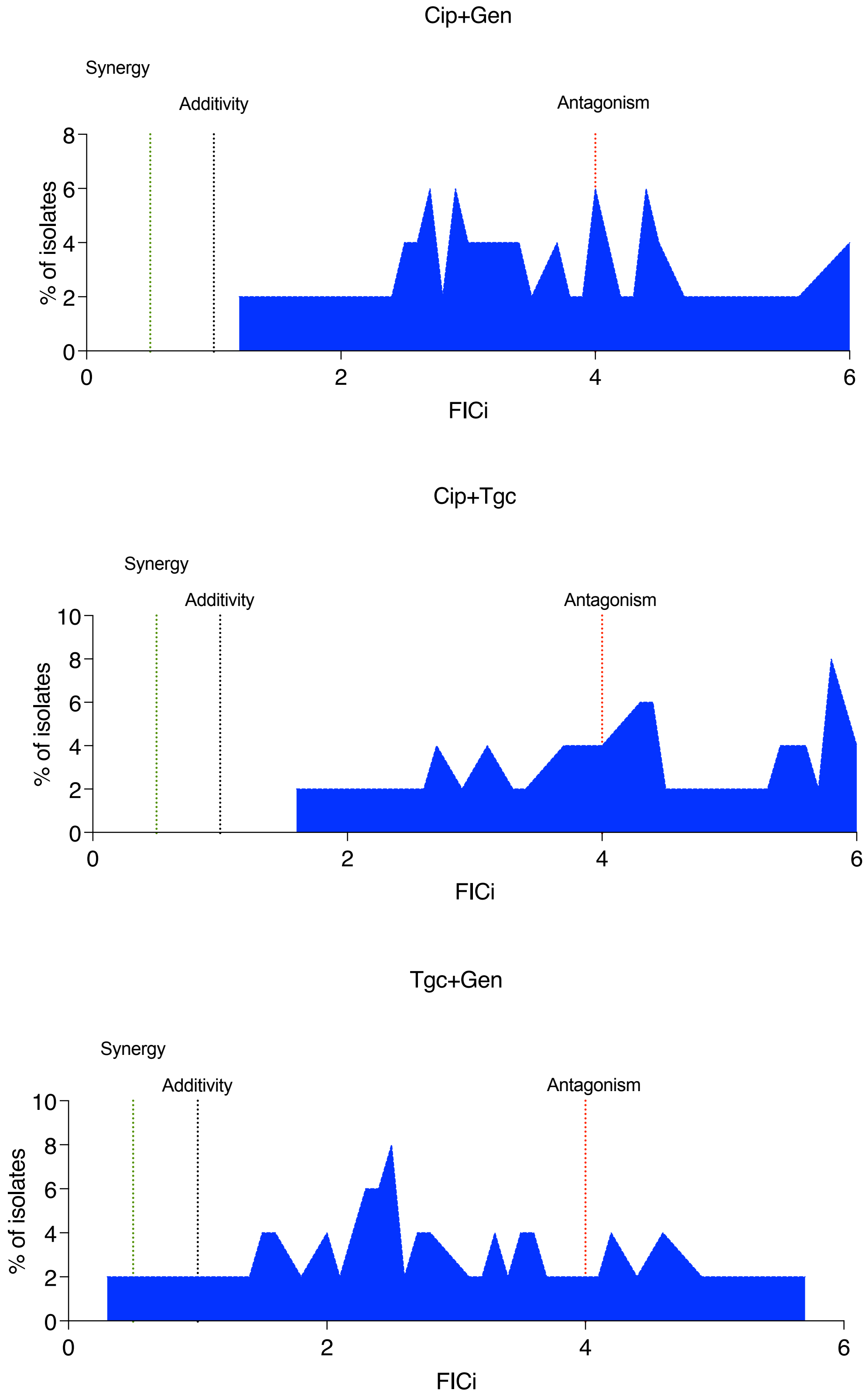
Cip+Gen
Synergy
Additivity
Antagonism
% of isolates
8
6
4
2
0
0
2
4
6
FICi
Cip+Tgc
Synergy
Additivity
Antagonism
% of isolates
10
8
6
4
2
0
0
2
4
6
FICi
Tgc+Gen
Synergy
Additivity
Antagonism
% of isolates
10
8
6
4
2
0
0
2
4
6
FICi

S7 Fig.

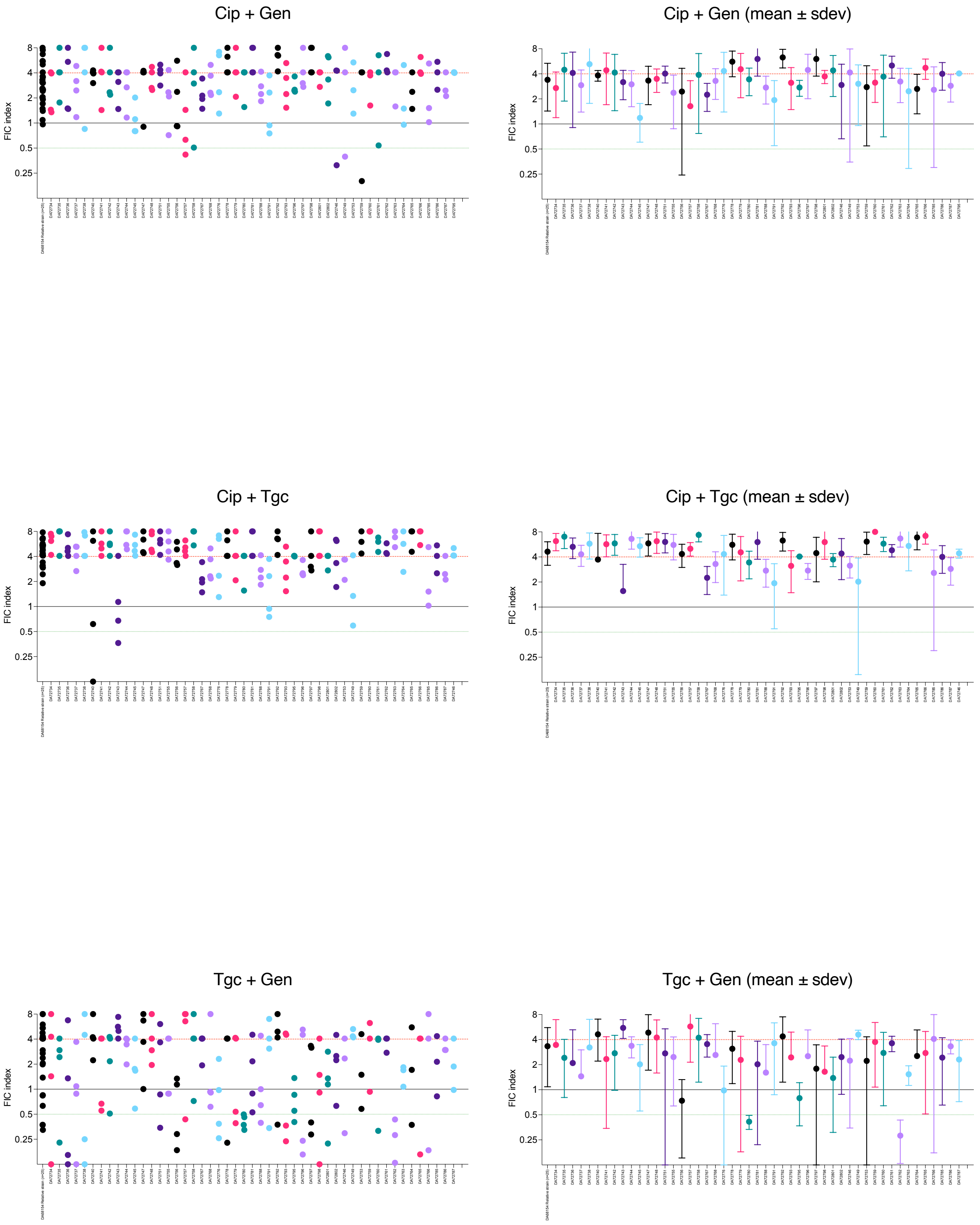
Cip + Gen
Cip + Gen (mean ± sdev)
Cip + Tgc
Cip + Tgc (mean ± sdev)
Tgc + Gen
Tgc + Gen (mean ± sdev)
FIC index
8
4
2
1
0.5
0.25

Supp Fig 8.

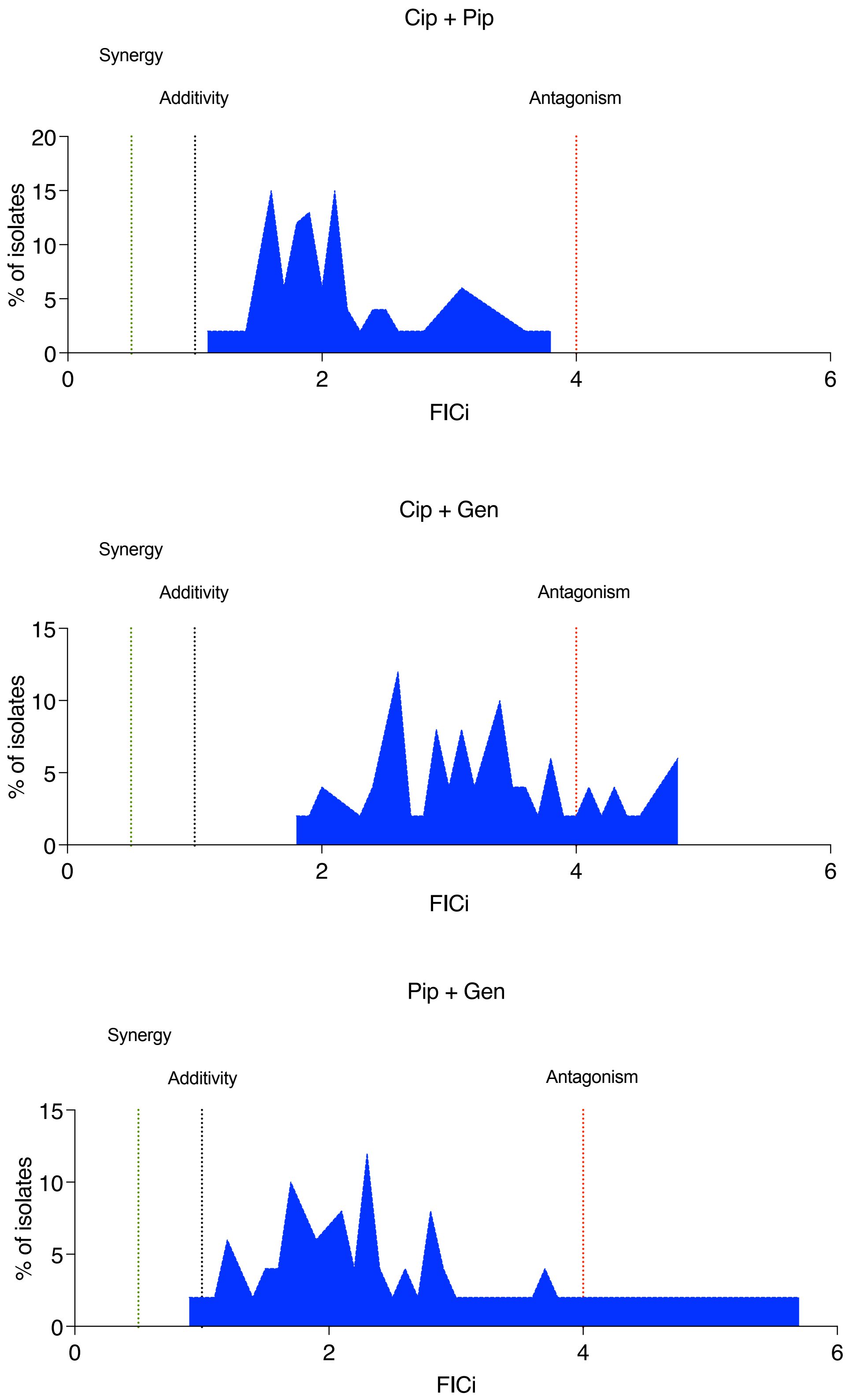
Cip + Pip
Synergy
Additivity
Antagonism
% of isolates
0
5
10
15
20
0
2
4
6
FICi
Cip + Gen
Synergy
Additivity
Antagonism
% of isolates
0
5
10
15
0
2
4
6
FICi
Pip + Gen
Synergy
Additivity
Antagonism
% of isolates
0
5
10
15
0
2
4
6
FICi

Supp Fig 9.

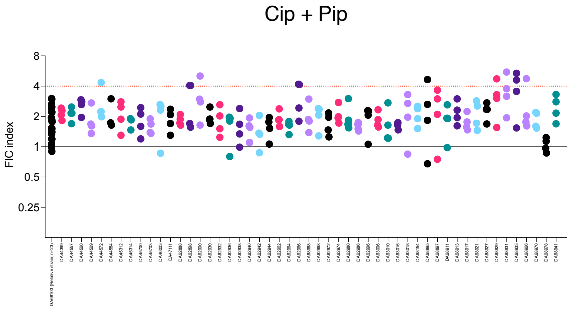
Cip + Pip
FIC index
8
4
2
1
0.5
0.25

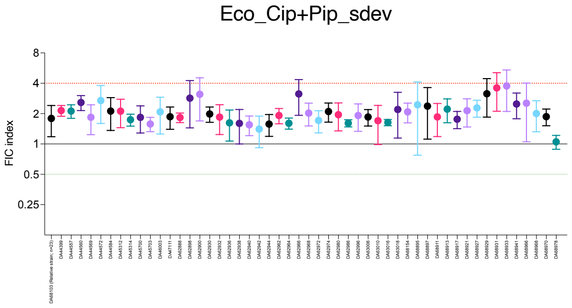
Eco_Cip+Pip_sdev
FIC index
8
4
2
1
0.5
0.25

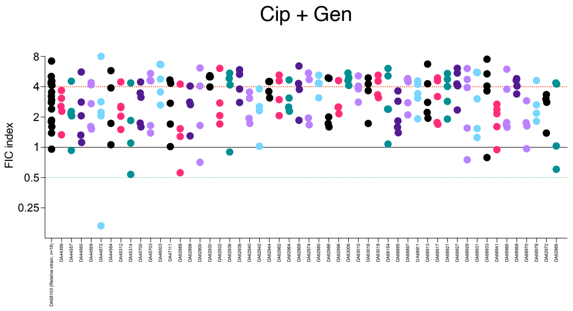
Cip + Gen
FIC index
8
4
2
1
0.5
0.25

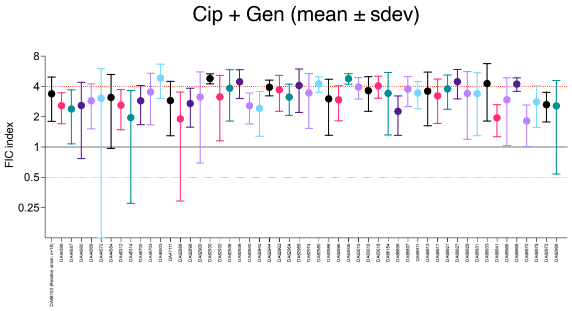
Cip + Gen (mean ± sdev)
FIC index
8
4
2
1
0.5
0.25

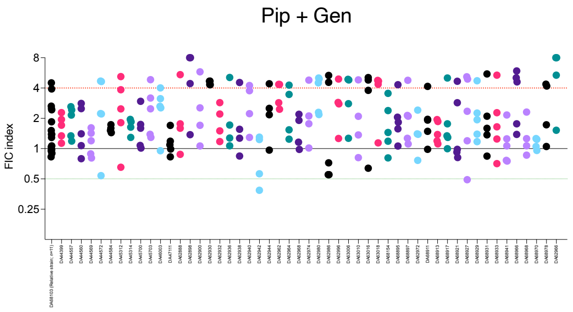
Pip + Gen
FIC index
8
4
2
1
0.5
0.25

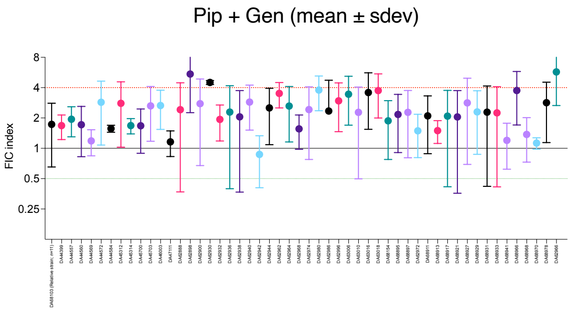
Pip + Gen (mean ± sdev)
FIC index
8
4
2
1
0.5
0.25

Supp Fig 10.

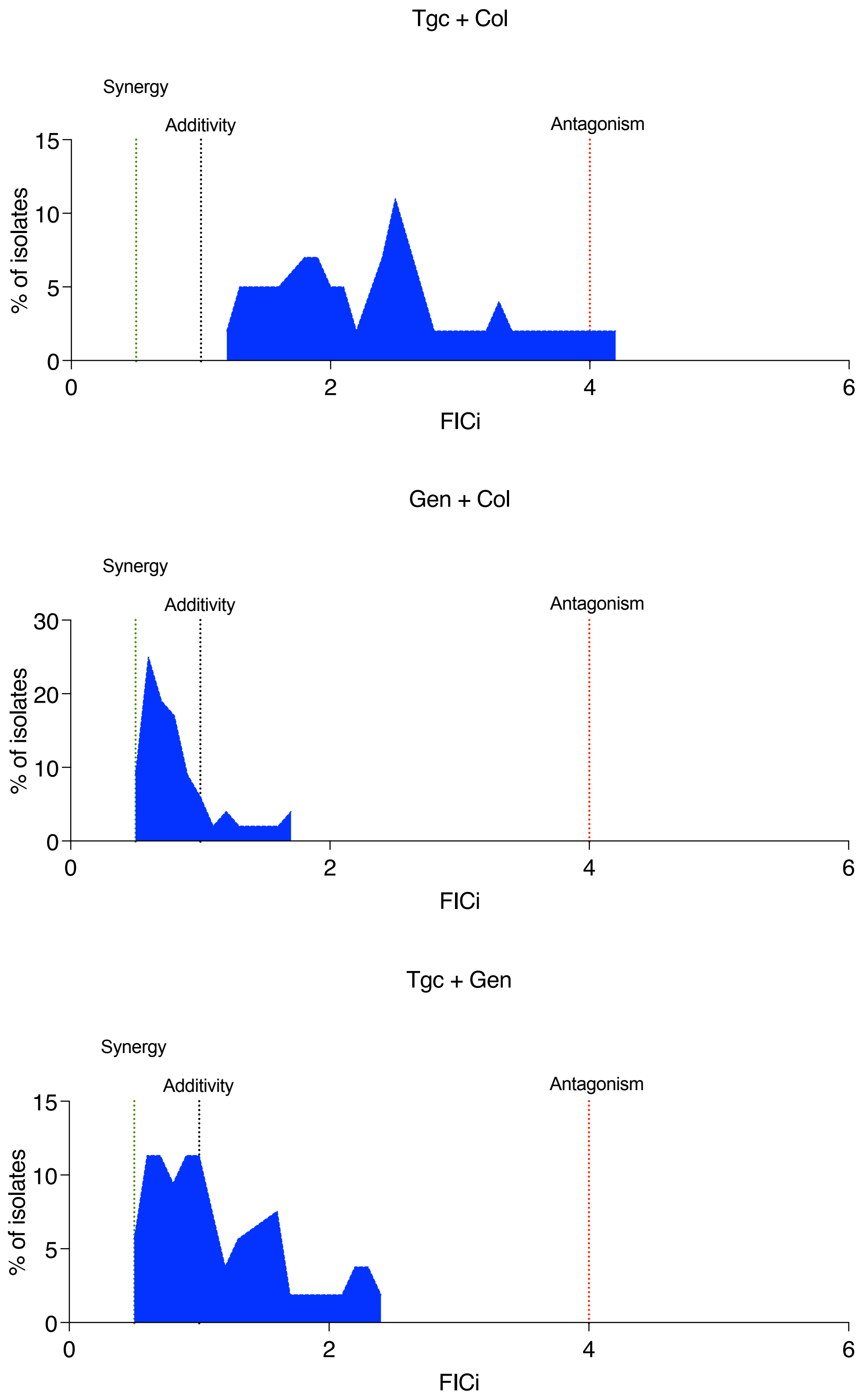
Tgc + Col
Synergy
Additivity
Antagonism
15
10
5
0
% of isolates
0
2
4
6
FICi
Gen + Col
Synergy
Additivity
Antagonism
30
20
10
0
% of isolates
0
2
4
6
FICi
Tgc + Gen
Synergy
Additivity
Antagonism
15
10
5
0
% of isolates
0
2
4
6
FICi

Supp Fig 11.

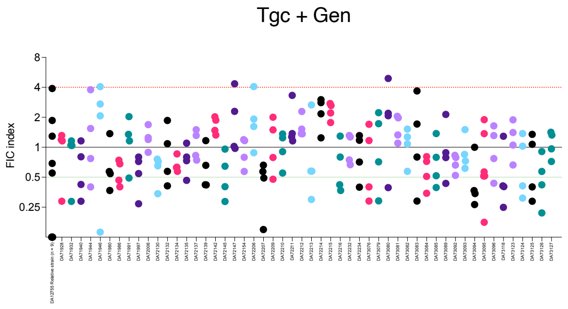
Tgc + Gen
FIC index
8
4
2
1
0.5
0.25

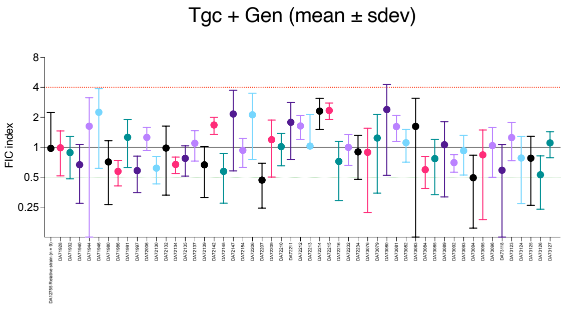
Tgc + Gen (mean ± sdev)
FIC index
8
4
2
1
0.5
0.25

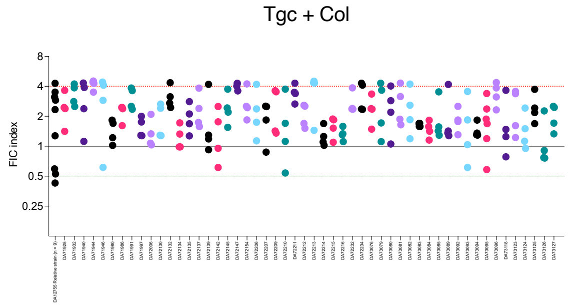
Tgc + Col
FIC index
8
4
2
1
0.5
0.25

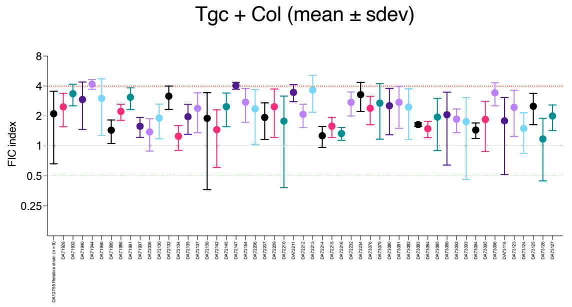
Tgc + Col (mean ± sdev)
FIC index
8
4
2
1
0.5
0.25

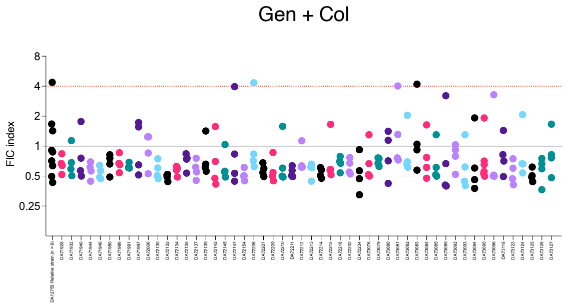
Gen + Col
FIC index
8
4
2
1
0.5
0.25

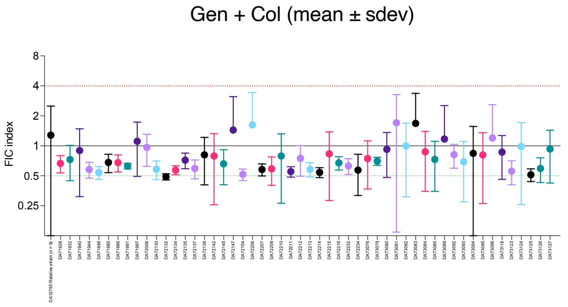
Gen + Col (mean ± sdev)
FIC index
8
4
2
1
0.5
0.25

Supp Fig 12.

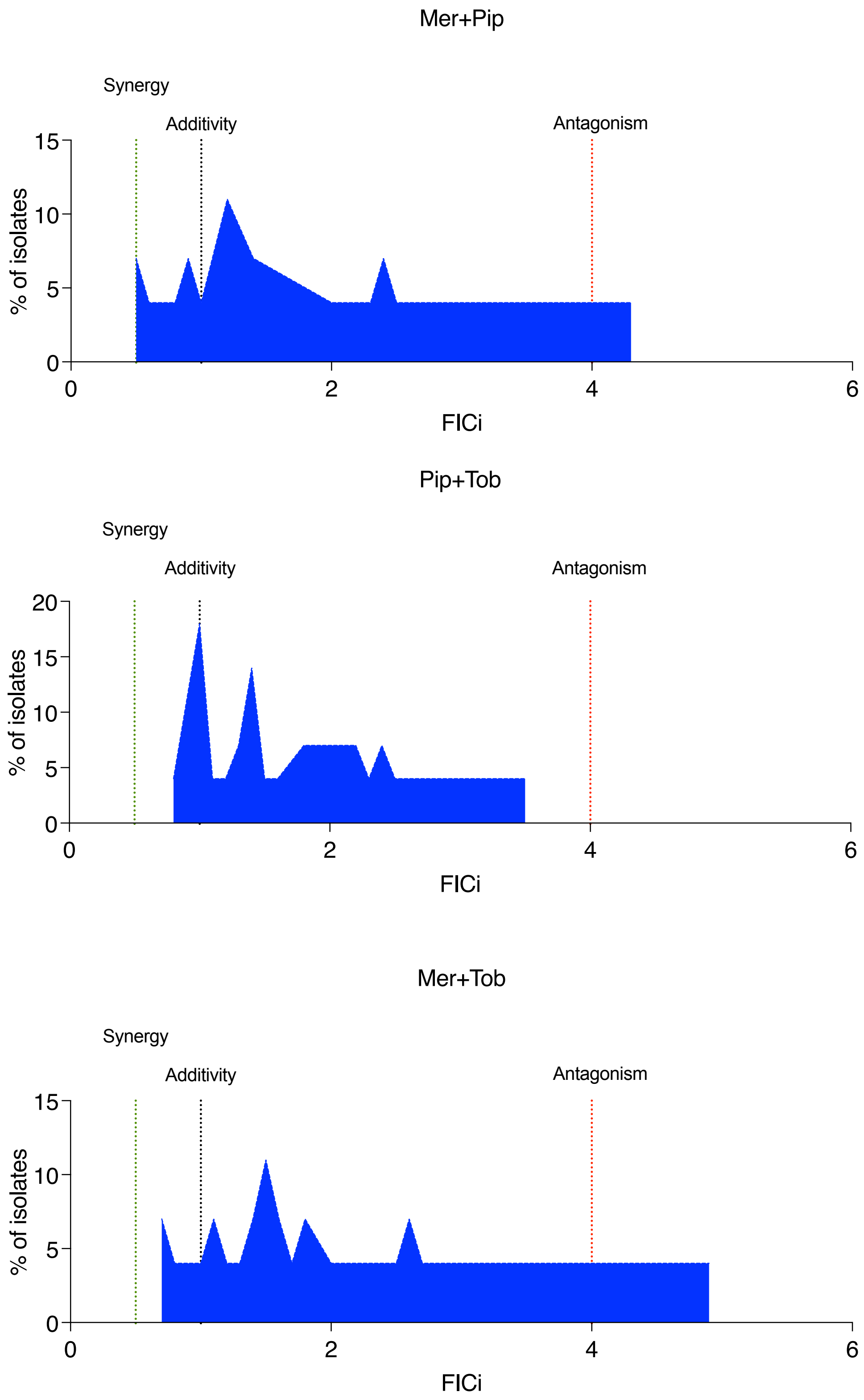

Mer+Pip
Synergy
Additivity
Antagonism
% of isolates
15
10
5
0
0
2
4
6
FICi
Pip+Tob
Synergy
Additivity
Antagonism
% of isolates
20
15
10
5
0
0
2
4
6
FICi
Mer+Tob
Synergy
Additivity
Antagonism
% of isolates
15
10
5
0
0
2
4
6
FICi

Supp Fig 13.

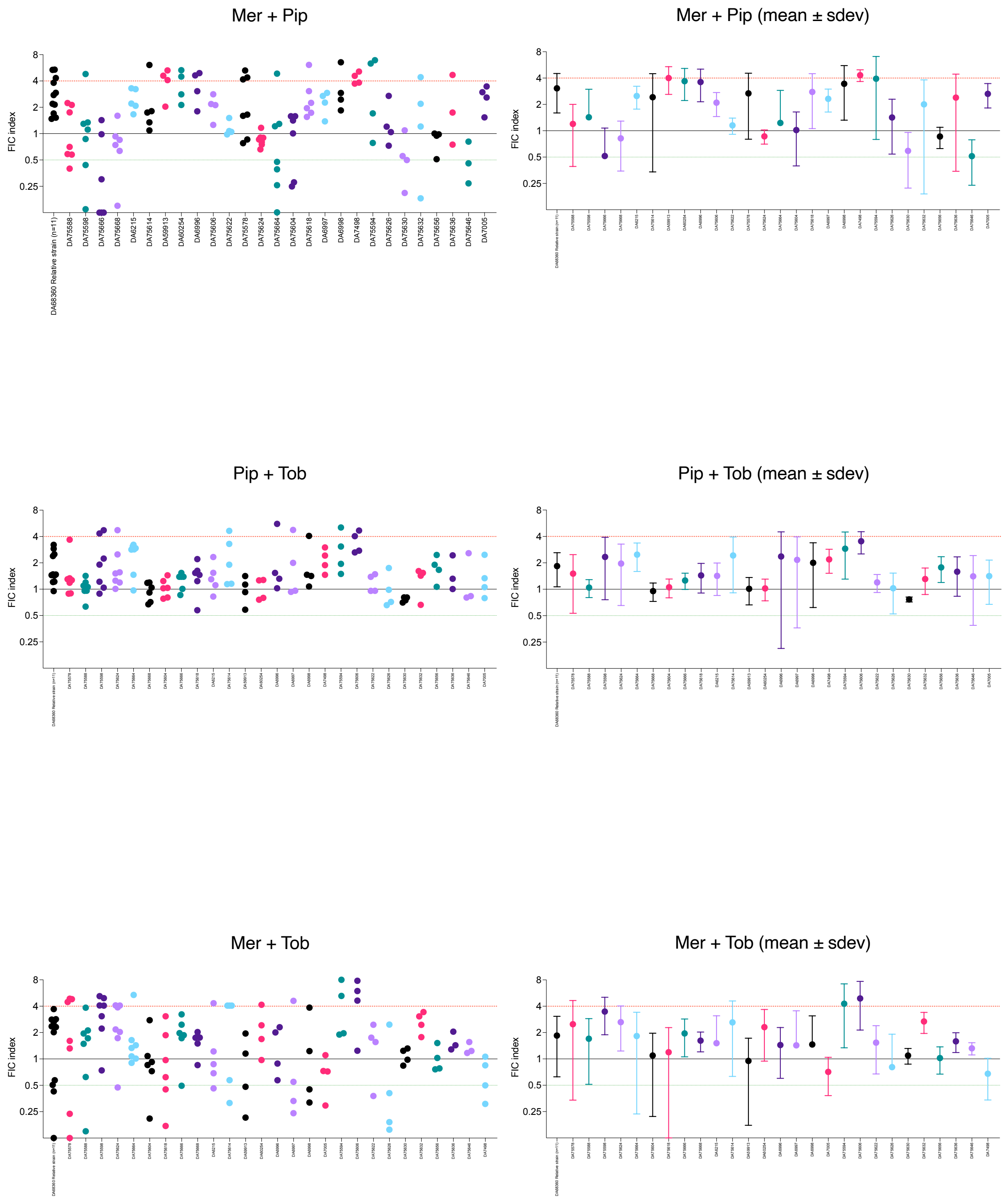
Mer + Pip
Mer + Pip (mean ± sdev)
Pip + Tob
Pip + Tob (mean ± sdev)
Mer + Tob
Mer + Tob (mean ± sdev)
FIC index
8
4
2
1
0.5
0.25
DA68360 Relative strain (n=11)
DA75588
DA75598
DA75666
DA75668
DA6215
DA75614
DA59913
DA60254
DA6996
DA75606
DA75622
DA75578
DA75624
DA75664
DA75604
DA75618
DA6997
DA6998
DA7498
DA75594
DA75626
DA75630
DA75632
DA75656
DA75636
DA75646
DA7005

Supp Fig 14.

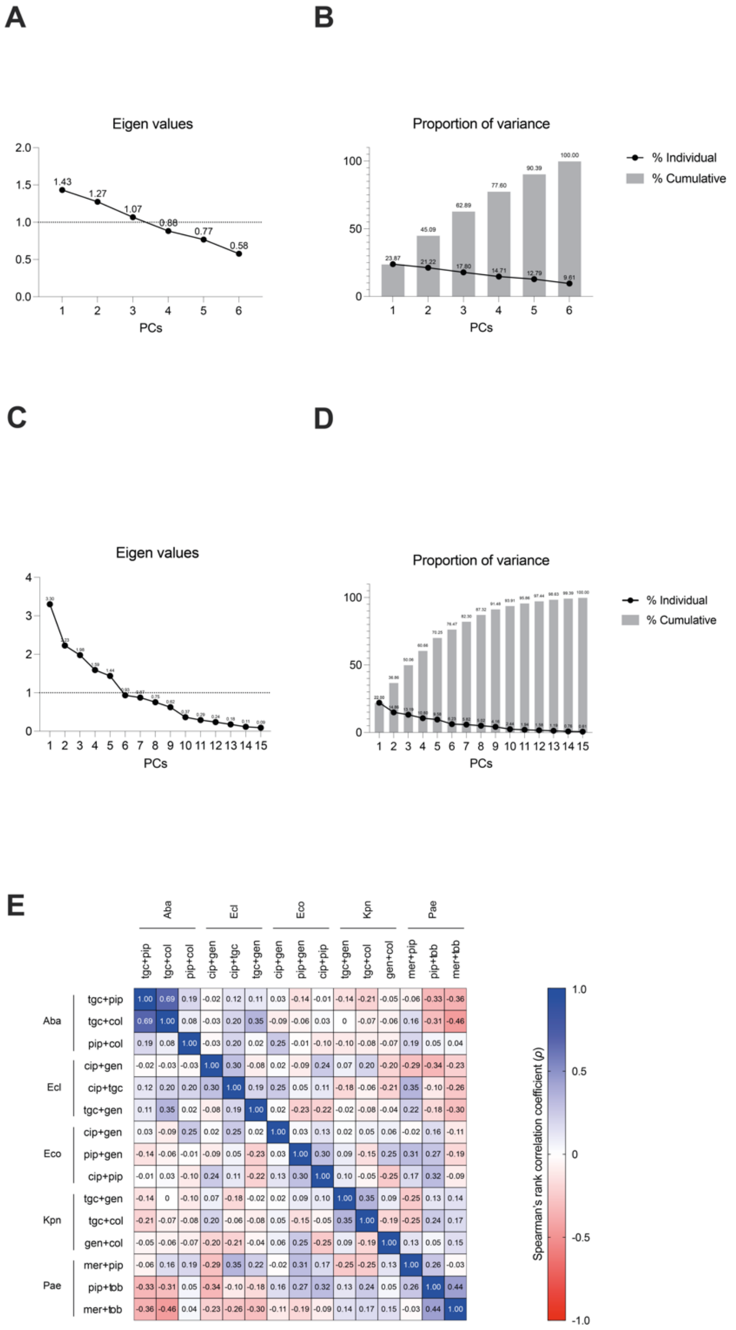
A
B
Eigen values
PCs
1.43
1.27
1.07
0.88
0.77
0.58
Proportion of variance
% Individual
% Cumulative
23.87
21.22
17.80
14.71
12.79
9.61
45.09
62.89
77.60
90.39
100.00
C
D
E
Aba
Ecl
Eco
Kpn
Pae
tgc+pip
tgc+col
pip+col
cip+gen
cip+tgc
tgc+gen
pip+gen
cip+pip
gen+col
mer+pip
pip+tob
mer+tob
Spearman's rank correlation coefficient (ρ)
